# Cervical dumbbell meningioma exhibiting distinct histopathological subtypes in intradural and extradural components: a case report

**DOI:** 10.1186/s12883-025-04550-5

**Published:** 2025-12-02

**Authors:** Chia-Yu Chen, Wei-Chuan Liao, Chia-Ing Jan

**Affiliations:** 1 Department of Neurosurgery, Kaohsiung Veterans General Hospital, Kaohsiung, Taiwan (R.O.C.); 2 Institute of Precision Medicine, National Sun-Yat-sen University, Kaohsiung, Taiwan (R.O.C.); 3 Department of Pathology, Kaohsiung Veterans General Hospital, Kaohsiung, Taiwan (R.O.C.); 4 School of Medicine, College of Medicine, National Sun-Yat-sen University, Kaohsiung, Taiwan (R.O.C.)

**Keywords:** Cervical spine, Dumbbell meningioma, Histopathological heterogeneity, Spinal tumour, Case report

## Abstract

**Background:**

Spinal dumbbell meningiomas are rare, accounting for only 4–5% of all spinal meningiomas. Histological heterogeneity within the same dumbbell lesion is even rarer, with all previously reported cases involving coexistence of a meningioma with either a schwannoma or a neurofibroma. To our knowledge, this is the first report of distinct meningioma subtypes occurring in separate anatomical compartments of a single dumbbell-shaped meningioma, a novel finding with important implications for surgical planning and outcomes.

**Case presentation:**

A 57-year-old man presented with progressive myelopathy and radiculopathy, including weakness and numbness of the left thumb and index finger, bilateral hyperreflexia, spasticity, and unsteady gait. Magnetic resonance imaging revealed a gadolinium-enhancing dumbbell-shaped tumour at the C5/6 level with a dural tail sign, consistent with an Eden type III spinal dumbbell tumour. The patient underwent two-stage microsurgical excision: initial posterior laminotomy and laminoplasty of C4–C6 with left C5/6 facetectomy for intradural tumour removal, followed by extraforaminal resection using a ventrolateral approach. Histopathology confirmed transitional and fibrous meningioma subtypes in the intradural and extraforaminal components, respectively. No fusion was performed after facetectomy, as postoperative alignment remained stable. Delayed C5–6 spondylolisthesis developed 2.5 years later and was successfully managed with cervical artificial disc replacement, restoring alignment and preserving motion. The patient achieved full neurological recovery with no recurrence at six-year follow-up.

**Conclusions:**

Dumbbell cervical meningiomas can present with exceptional histopathological heterogeneity, with distinct transitional and fibrous subtypes identified in the intradural and extraforaminal components, respectively. The excellent long-term outcomes achieved with a tailored two-stage surgical approach highlight the importance of individualised strategies for treating these rare and complex lesions. Recognising the potential histological diversity of dumbbell-shaped meningiomas is essential for effective surgical planning and optimisation of patient outcomes.

**Supplementary Information:**

The online version contains supplementary material available at 10.1186/s12883-025-04550-5.

## Highlights

This is the first reported case of a cervical dumbbell meningioma exhibiting two distinct WHO grade I subtypes—transitional and fibrous—located in separate anatomical compartments of a single tumour.

A two-stage microsurgical strategy, combining a posterior approach followed by ventrolateral resection, achieved gross-total resection while preserving neurological function.

The patient achieved complete neurological recovery and has remained disease-free for six years after surgery.

**Supplementary Information:**

The online version contains supplementary material available at 10.1186/s12883-025-04550-5.

## Background

Spinal dumbbell meningiomas are rare, accounting for only 4–5% of all spinal meningiomas [1, 2]. They develop a characteristic dumbbell configuration by extending from the intradural compartment through the intervertebral foramen into the extradural and extraforaminal spaces [3]. This growth pattern presents unique anatomical and surgical challenges, making complete resection difficult [1]. Histological heterogeneity within a single dumbbell lesion is exceedingly rare, with all previously reported cases involving the coexistence of a meningioma with either a schwannoma or a neurofibroma [1]. Owing to the limited number of such reports, the clinical features and optimal surgical strategies remain unclear. To our knowledge, this is the first documented case of distinct meningioma subtypes occurring in separate anatomical compartments of a single dumbbell-shaped meningioma, successfully treated with a two-stage approach combining posterior and subsequent ventrolateral cervical procedures. This case report has been reported in line with the SCARE checklist [4].

## Case presentation

**Fig. 1:**
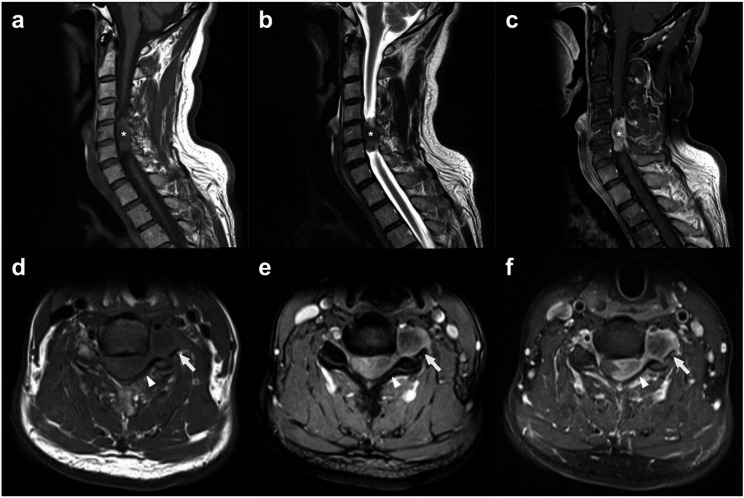
Preoperative cervical spine MRI showing a C5–6 dumbbell-shaped meningioma with both intradural and foraminal components. **a** Sagittal T1-weighted image demonstrates an intradural extramedullary mass (asterisk) that is isointense to the spinal cord at the left C5–6 level. **b** Sagittal T2-weighted image shows a heterogeneously iso- to hyperintense lesion (asterisk). **c** Sagittal post-contrast T1-weighted image reveals a well-demarcated, intensely and nearly homogeneously enhancing mass (asterisk) with a dural tail sign. **d** Axial T1-weighted image demonstrates a dumbbell-shaped lesion, isointense to the cord, extending from the intradural compartment (arrowhead) through the left C5–6 neural foramen (arrow). **e** Axial T2-weighted image shows a left C5–6 dumbbell-shaped mass encroaching on the left side of the spinal canal (arrowhead) with rightward displacement of the spinal cord, extending through the left intervertebral foramen (arrow). **f** Axial post-contrast T1-weighted image shows strong, homogeneous enhancement of the dumbbell-shaped mass, which extends through the left C5–6 foramen (arrows) and causes marked rightward displacement of the cervical spinal cord (arrowhead). No flow voids are present, indicating a non-hypervascular lesion

A 57-year-old man presented with progressive cervical myelopathy and radiculopathy, characterized by MRC Grade 4 strength of the left grasp, numbness over the left thumb and index finger (C6 dermatome), positive Hoffman sign, positive Lhermitte and Spurling signs, hyperreflexia and spasticity in both upper and lower extremities, and an unsteady tandem gait. Plantar responses were bilaterally negative, and no autonomic involvement was noted. Preoperative magnetic resonance imaging (MRI) revealed a well-demarcated, dumbbell-shaped tumour at the left C5–6 level, extending from the intradural extramedullary compartment to the extraforaminal space (Fig. 1). The lesion appeared isointense to the spinal cord on T1-weighted images and heterogeneously iso- to hyperintense on T2-weighted sequences. Post-gadolinium T1-weighted images demonstrated strong, nearly homogeneous enhancement with a dural-tail sign. The mass caused compression and marked rightward displacement of the cervical cord without intramedullary signal change. No intratumoural or peritumoural flow voids were identified, consistent with a non-hypervascular meningioma.

The patient underwent a staged microsurgical excision. The first procedure involved a left posterior laminotomy and laminoplasty of C4–C6, along with a left C5/6 facetectomy, to remove the intraspinal portion of the tumour while minimising spinal cord injury. A second surgery via a ventrolateral cervical approach was performed 6 months later to excise the extraforaminal portion, following careful neurolysis and exploration of the C5 and C6 nerve roots. The tumour was completely resected (Simpson grade 2). Notably, the tumour was soft to rubbery in the intradural compartment and firm in the extraforaminal component.

**Fig. 2:**
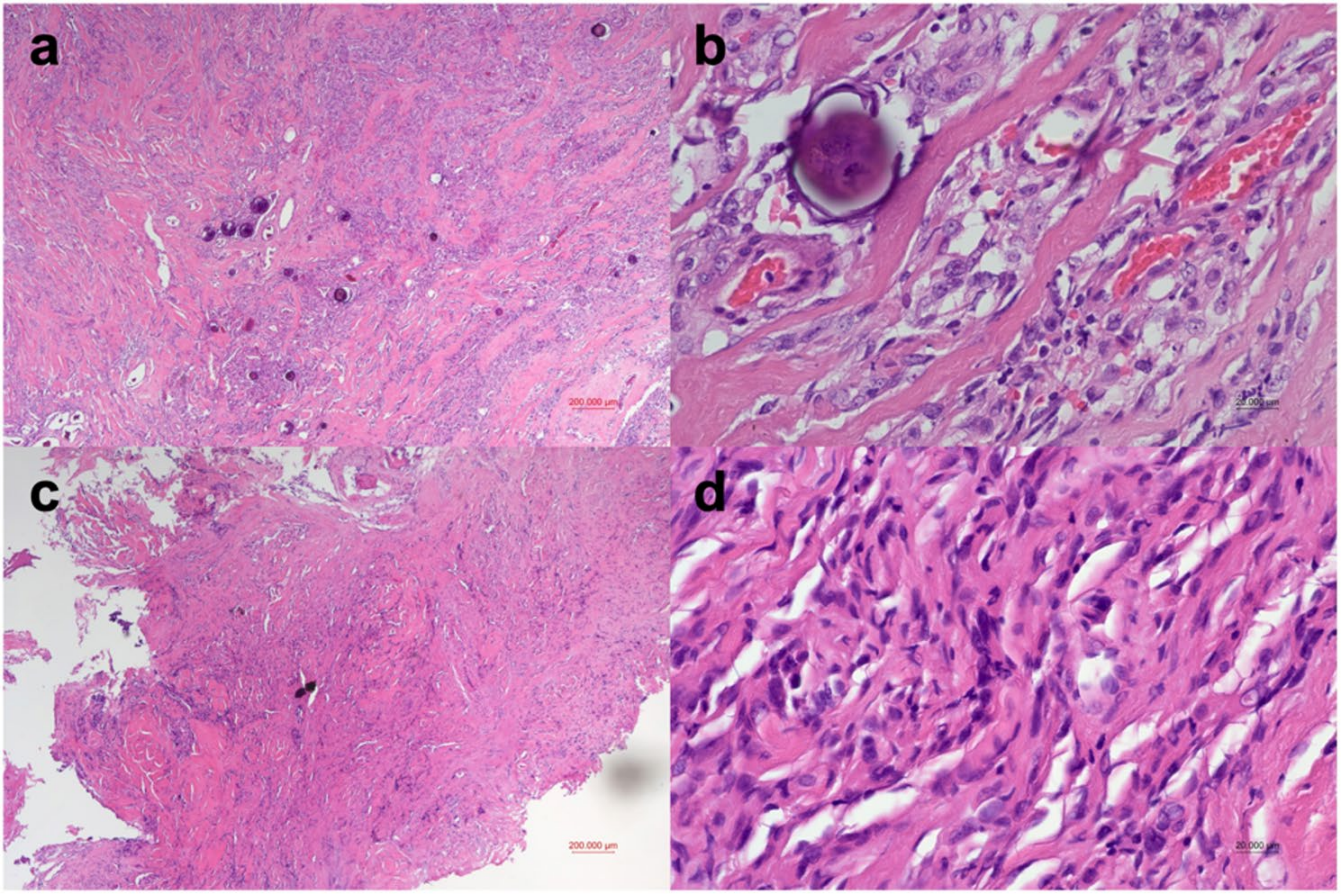
Histopathological features (H&E staining) of the intradural and extraforaminal tumour components. **a**–**b** Intradural component resected during the first-stage surgery, diagnosed as transitional meningioma (WHO grade I). **a** Low-power H&E (×40) showing mixed meningothelial and fibrous areas with whorl formation and psammoma bodies. **b** High-power H&E (×400) highlighting tight whorls and prominent psammoma bodies. **c**–**d** Extraforaminal component resected during the second-stage surgery, diagnosed as fibrous meningioma (WHO grade I). **c** Low-power H&E (×40) demonstrating densely collagenous stroma and fascicular spindle-cell arrangement. **d** High-power H&E (×400) showing elongated spindle cells with abundant collagen fibres, lacking whorls or psammoma bodies

**Fig. 3:**
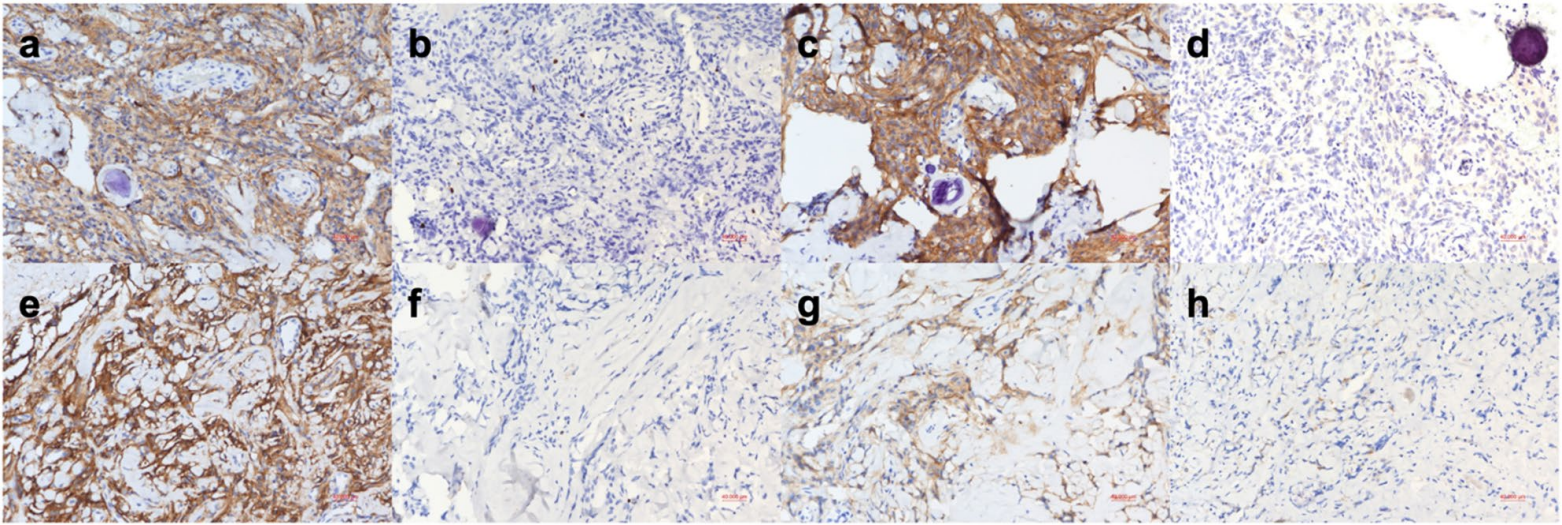
Immunohistochemical profiles of EMA, Ki-67, SSTR2A, and STAT6 expression in the intradural and extraforaminal components. **a**–**d** Intradural component resected during the first surgery, diagnosed as transitional meningioma (WHO grade I). **a** EMA (200×) shows diffuse membranous positivity with whorled architecture, characteristic of transitional meningioma with cohesive meningothelial differentiation. **b** Ki-67 (200×) demonstrates scattered, focally positive nuclei with an estimated labelling index of ~2–3%, indicating low proliferative activity. **c** SSTR2A (200×) shows diffuse, strong membranous positivity highlighting whorled cellular arrangements, confirming meningothelial differentiation with robust somatostatin receptor expression. **d** STAT6 (200×) shows an absence of nuclear staining with only faint background reactivity, consistent with meningioma and excluding solitary fibrous tumor/hemangiopericytoma (SFT/HPC). **e**–**h** Extraforaminal component resected during the second surgery, diagnosed as fibrous meningioma (WHO grade I). **e** EMA (200×) shows coarse, patchy membranous to cytoplasmic positivity along spindle cells and collagen bundles, consistent with fibrous meningioma and indicating reduced epithelial differentiation. **f** Ki-67 immunostaining (200×) demonstrates very sparse nuclear staining, with most areas showing virtually no proliferative activity, and an estimated labelling index of <1%, indicating low proliferative activity. **g** SSTR2A (200×) demonstrates weak, focal, and patchy membranous positivity, reflecting attenuated receptor expression consistent with fibroblastic differentiation. **h** STAT6 (200×) is negative, with minimal background staining, confirming the absence of STAT6 expression and supporting the diagnosis of fibrous meningioma rather than a fibrous-type SFT/HPC

The tumour was classified as Eden type III, involving intraspinal, foraminal, and paraspinal (extraforaminal) components [5]. Histopathological examination revealed distinct morphologies between the intradural and extraforaminal tumour components. The intradural specimen obtained during the first-stage surgery was diagnosed as transitional meningioma (WHO grade I), exhibiting mixed meningothelial and fibrous areas with prominent whorl formation and psammoma bodies. In contrast, the extraforaminal specimen from the second-stage surgery demonstrated fibrous meningioma (WHO grade I), composed of densely collagenous stroma with fascicular spindle-cell proliferation lacking whorls or psammoma bodies, confirming two histological subtypes within the same lesion (Fig. 2). Immunohistochemically, both components showed diffuse EMA and SSTR2A positivity, negative STAT6 staining, and low proliferative activity (Ki-67 labelling index < 3%), consistent with benign meningioma and excluding solitary fibrous tumour/hemangiopericytoma. The extraforaminal component exhibited relatively weaker and patchier EMA and SSTR2A expression, in keeping with fibrous differentiation (Fig. 3). Both specimens were independently reviewed by two neuropathologists.

The postoperative course was uneventful, with no new neurological deficits observed. The patient’s left-hand grip strength gradually improved within 3 months, while tandem gait and spasticity recovered more slowly but progressively, returning to near-normal by 5–6 months postoperatively. He was able to resume normal daily activities.

**Fig. 4:**
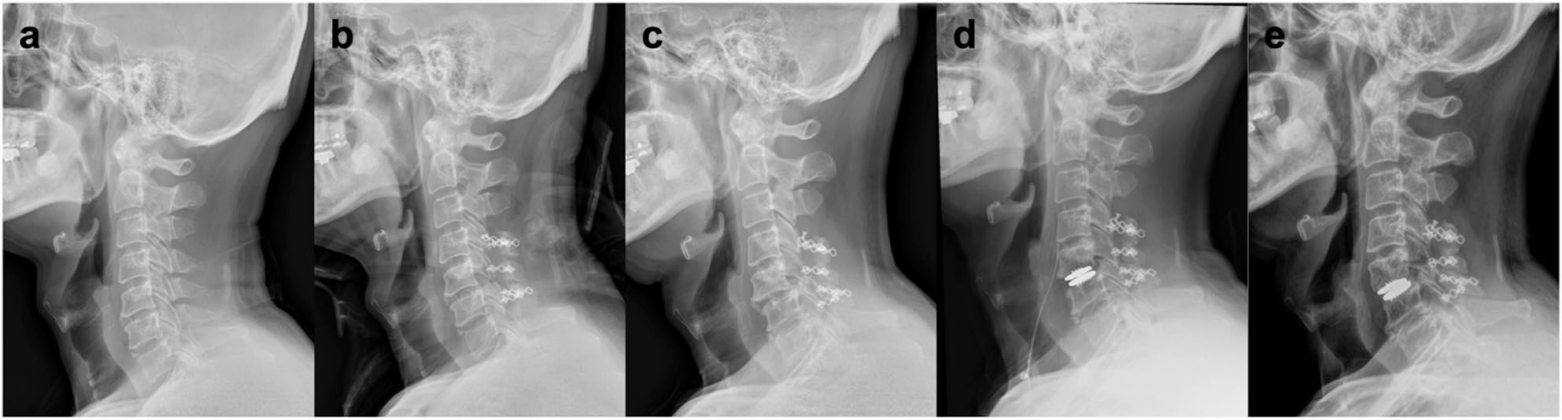
Sequential lateral cervical spine radiographs demonstrating postoperative evolution following staged resection of a C5–6 dumbbell meningioma. **a** Preoperative radiograph showing preserved cervical alignment. **b** Early postoperative image after the second-stage ventrolateral resection with right C5–6 facetectomy, demonstrating stable alignment without instrumentation. **c** Follow-up radiograph at 2.5 years showing progressive C5–6 spondylolisthesis, consistent with delayed instability after facetectomy. **d** Post–artificial disc replacement (ADR) radiograph demonstrating restoration of disc height and cervical alignment. **e** Follow-up radiograph at 3 years after ADR showing a stable implant

**Fig. 5:**
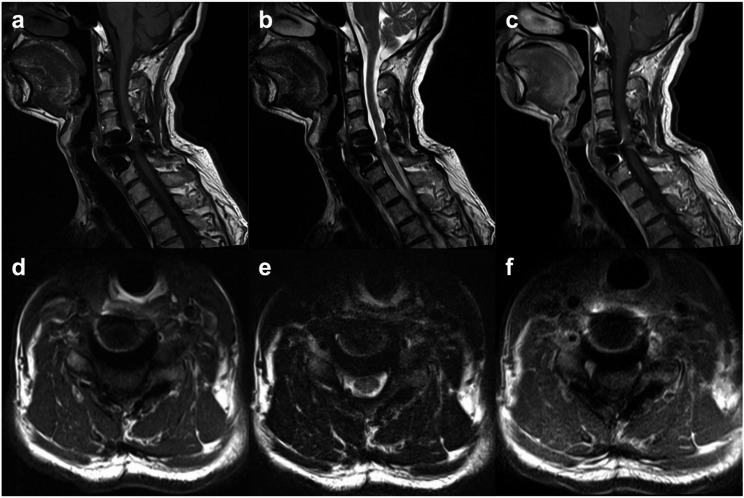
Follow-up MRI at 6 years. **a**–**c** Sagittal T1-weighted (**a**), T2-weighted (**b**), and post-contrast T1-weighted (**c**) images show no evidence of residual or recurrent intradural tumour at the C5–6 level. The spinal cord is well decompressed, and the dural contour remains smooth without abnormal enhancement. A susceptibility artifact from the C5–6 artificial disc replacement is visible at the operative level. **d**–**f** Corresponding axial T1-weighted (**d**), T2-weighted (**e**), and post-contrast T1-weighted (**f**) images confirm complete tumour removal and resolution of cord compression, with no enhancing lesion in the intradural or foraminal region

No fusion was performed initially following the C5–6 facetectomy, as both preoperative and postoperative images demonstrated stable alignment with preservation of the contralateral facet and posterior tension band. Spinal stability was subsequently monitored through serial radiographs, which revealed progressive C5–6 spondylolisthesis 2.5 years postoperatively. The patient later underwent cervical artificial disc replacement (ADR) at the same level, achieving restoration of disc height and alignment (Fig. 4). Follow-up radiographs three years after ADR demonstrated a stable implant. ADR was selected over fusion as a motion-preserving strategy for mild, delayed instability in a relatively young patient with preserved contralateral facet and posterior elements. There was no evidence of tumour recurrence at the 6-year follow-up (Fig. 5). Written informed consent for the publication of this case report and the accompanying images was obtained from the patient.

### Discussion and conclusions

This case illustrates a unique cervical dumbbell meningioma with distinct histological subtypes: intradural transitional meningioma and extraforaminal fibrous meningioma. Complete tumour removal was achieved through a two-stage approach utilising posterior laminotomy and laminoplasty, followed by a ventrolateral cervical procedure. The extradural tumour component was notably firm, making dissection challenging. A left C5/6 facetectomy was required to ensure adequate surgical exposure and minimise the risk of spinal cord injury. At the 6-year follow-up, the patient had fully returned to normal life and work, with no recurrence or sustained neurological recovery. To our best knowledge, this is the first report of a distinct meningioma subtype in separate anatomical compartments of a dumbbell-shaped cervical meningioma.

Meningiomas are a heterogeneous group of tumours that most commonly arise in the intradural extramedullary compartment of the spinal cord [6]. Dumbbell-shaped meningiomas are rare, with a limited number of cases reported in the literature [1, 2]. Unlike conventional spinal meningiomas which may include higher-grade variants [6], all reported spinal dumbbell meningiomas are uniformly benign, being WHO grade I [1, 2]. In the current case, histopathology revealed a transitional meningioma in the first operation and a fibrous meningioma in the second operation. These findings are uncommon as there are virtually no reports of different meningioma subtypes in separate anatomical compartments. To date, only 13 single dumbbell-shaped spinal tumours containing histologically distinct components—typically meningioma coexisting with schwannoma or neurofibroma—have been reported [7–15] (Table 1). In contrast, this case features two distinct meningioma subtypes (transitional and fibrous) within a single dumbbell lesion.

**Table 1:** Summary of reported cases of spinal dumbbell-shaped tumors with concurrent histological heterogeneity

Authors/ Year	Age/Sex	Levels	Surgical Approach	Staging	Histopathology	Extent of resection	Outcomes
Present case (2025)	57/M	C5–6	Posterior (C4–6 laminotomy and laminoplasty with left C5–6 facetectomy) + ventrolateral approach	Two-stage	Intradural transitional meningioma (WHO I) + extraforaminal fibrous meningioma (WHO I)	GTR (Simpson grade II, complete resection of both components)	Complete neurologic recovery; disease-free at 6-year follow-up
Zheng et al. (2023) [7] – Case 1	53/M	C2	Posterior (details NR)	Single-stage	Intradural meningioma (WHO I) + extradural schwannoma	GTR (intact subcapsular resection)	Good neurologic recovery; no neurologic deficit
Zheng et al. (2023) [7] – Case 2	47/F	C2	Posterior (details NR)	Single-stage	Intradural meningioma (WHO I) + extradural schwannoma	GTR (intact subcapsular resection)	Improved, ambulatory with walker at 6 months
Zheng et al. (2023) [7] – Case 3	55/M	C2	Posterior (details NR)	Single-stage	Intradural meningioma (WHO I) + extradural schwannoma	GTR (intact subcapsular resection)	Neurologic improvement; no neurologic deficit at 2.5-year follow-up
Zheng et al. (2023) [7] – Case 4	61/M	C2	Posterior (details NR)	Single-stage	Intradural meningioma (WHO I) + extradural schwannoma	GTR (intact subcapsular resection)	Neurologic improvement; no neurologic deficit
Zhan et al. (2019) [8]	47/F	C1–C2	Posterior (left suboccipital craniectomy + C1–2 hemilaminectomy)	Single-stage	Intradural neurofibroma + extradural meningioma (WHO I)	GTR (coagulated and resected the adjacent dura mater)	Good neurologic recovery; no recurrence at 3-month follow-up
Matsuda et al. (2018) [9] – Case 1	53/M	C2	Posterior (C1–2 laminectomy)	Single-stage	Intradural meningioma (WHO I) + extradural schwannoma	GTR (subcapsular extradural + total intradural removal)	Good neurologic recovery; no neurologic deficit
Matsuda et al. (2018) [9] – Case 2	70/M	C2	Posterior (right suboccipital craniectomy + C1–2 hemilaminectomy)	Single-stage	Intradural meningioma (WHO I) + extradural schwannoma	GTR	Good neurologic recovery
Liebelt et al. (2016) [10]	58/M	C1–Foramen Magnum	Posterior (right suboccipital craniectomy + C1 hemilaminectomy)	Single-stage	Intradural meningioma (WHO I) + extradural schwannoma	GTR	Good neurologic condition; no new deficits; discharged POD 2
Oichi et al. (2015) [11]	64/M	C2	Posterior (C1 hemilaminectomy)	Single-stage	Intradural meningioma (WHO I) + extradural schwannoma	GTR (coagulated and resected the adjacent dura mater)	Occipital pain resolved; no recurrence at 6-month follow-up
Chen et al. (2014) [12]	72/F	C3–C4	Posterior (C3–4 laminectomy + posterior instrumentation)	Single-stage	Intradural meningioma (WHO I) + extradural neurofibroma	GTR (extradural removal with C3 root sacrifice + total intradural removal)	Full neurologic recovery; no neurologic deficit; no recurrence at 12-month follow-up.
Nakamizo et al. (2012) [13]	49/M	C2	Posterior (C1 laminoplastic laminotomy + C2 partial laminectomy)	Single-stage	Intradural meningioma (WHO I) + extradural schwannoma	GTR (coagulated the dura mater adjacent to the intradural tumor and resected the dura mater around the C2 root exits)	Full neurologic recovery; sensory improvement, no complications
Ogihara et al. (2003) [14]	54/F	C4–C5	Posterior (details NR)	Single-stage	Intradural meningioma (WHO I) + extradural schwannoma	GTR (coagulated the dura adjacent to the intradural meningiomas)	Sensory and gait improvement; transient right deltoid/biceps weakness with partial recovery by 24 months
Hokari et al. (2002) [15]	59/F	C1–2	Posterior (details NR)	Single-stage	Intradural meningioma (WHO I) + extradural neurofibroma	NR	Symptoms resolved

*GTR* Gross-total resection, *NR* Not reported, *POD* Postoperative day, *WHO* World Health Organization tumor grade, *M* Male, *F* Female

These composite or collision tumours pose considerable diagnostic challenges. On preoperative MRI, they often mimic a single dumbbell-shaped mass, obscuring their underlying histological diversity. Intraoperative discrepancies—such as marked differences in tumour colour, texture, or firmness between compartments—should alert the surgeon to the possibility of histologically distinct components and prompt separate sampling to avoid underdiagnosis of biphasic or mixed lesions [10].

Histological heterogeneity itself did not correlate with adverse outcomes. Postoperative neurological outcome was generally favourable following gross-total resection (GTR) (Table 1). Surgical management, however, requires balancing the objective of complete resection with preservation of neurological function. Reported complications include nerve root sacrifice [12], partial motor weakness [14], and the need for dural repair following durotomy to prevent cerebrospinal fluid (CSF) leakage [11]. Instrumented stabilisation may be warranted when extensive bone removal compromises spinal stability [12].

Several theories to explain the rare occurrence of concurrent histologically distinct tumours at the same spinal level have been proposed. The tumour microenvironment theory suggests that varying mechanical pressures and anatomical constraints promote the development of different tumour lineages. The intradural compartment cushioned by the CSF experiences lower mechanical stress and contains neural-specific matrix components, whereas the extraforaminal compartment is subject to greater mechanical forces and is characterised by a denser collagen-rich extracellular matrix [16]. Vascular and metabolic differences further contribute to compartmental heterogeneity. Intradural regions are protected by the blood-brain barrier and exhibit controlled permeability, whereas extraforaminal areas have more permeable, fenestrated vessels, resulting in differences in oxygen and nutrient delivery and potentially activating hypoxia-inducible factors (e.g. HIF-1α) [16].

Neural microenvironments also differ. While intradural tumours are influenced by central nervous system (CNS)-specific growth factors and cell adhesion molecules, extraforaminal tumours are exposed to peripheral neurotrophic factors and Schwann cell-derived cues [16]. Stromal differences are reflected in CNS-specific fibroblasts in the intradural space and peripheral fibroblasts in the extraforaminal regions, each of which contributes to unique paracrine signals. Additionally, the local microenvironment may be altered by the presence of a primary tumour, which can induce reactive changes in adjacent meningothelial cells and drive meningioma heterogeneity, particularly near the nerve root outlets [7, 8].

Meanwhile, the common progenitor cell theory posits that both tumours originate from a shared mesenchymal progenitor cell that differentiates along multiple lines in response to specific genetic or environmental cues, particularly when tumours are closely intermingled or exhibit mixed growth patterns [7, 8]. The shared oncogenic stimulus theory proposes that neoplastic transformation of different cell types may occur simultaneously at the same site owing to a common carcinogenic factor, such as prior radiation exposure [8]. Finally, the coincidence theory suggests that simultaneous development of the two tumour types may be incidental. However, an increasing number of reported cases suggests that coincidence alone is unlikely to explain all occurrences [7, 8].

In the current case, the transitional meningioma appeared macroscopically elastic, whereas the fibrous meningioma was firm and adhered to the cord, roots, and surrounding structures. The physical consistency of meningiomas varies according to the subtype. Fibroblastic meningiomas are consistently the firmest subtype. They are associated with a significantly lower GTR rate compared to softer tumours (43% vs. 77%) [17, 18], while operative times tend to be modestly longer (by ~ 20–30 min on average), though this difference is not statistically significant. Their abundant collagen makes them ‘hard’, necessitating sharp dissection and high-power ultrasonic aspiration. In contrast, meningothelial and transitional meningiomas are “rubbery” and easier to handle; they can often be internally debulked with suction and their capsules folded during dissection [18]. These characteristics fundamentally impact the surgical strategy. Soft tumours can be treated using minimally invasive techniques with en bloc resection when anatomically feasible. In contrast, firm tumours typically require appropriate exposure with piecemeal removal.

The posterior approach remains standard for most spinal meningiomas; however, complex lesions may require combined or staged strategies [19]. In our case, a two-stage approach was selected for a dumbbell-shaped Eden type III meningioma characterised by substantial extraforaminal extension, firm fibrous consistency, challenging dural attachments, and proximity to the vertebral artery—features that increased the risk of traction injury to critical cervical nerve roots. The need for extensive bone resection, wide exposure through distinct surgical corridors, patient repositioning, and prolonged operative time further supported a staged strategy.

Among previously reported cervical dumbbell meningiomas with histological heterogeneity, most were located at the high cervical levels (C1–C2), with only two exceptions (Table 1). Chen et al. described a C3–C4 tumour that achieved GTR at the cost of C3 nerve root sacrifice but without persistent deficit [12], whereas Ogihara et al. reported a C4–C5 lesion achieving GTR with postoperative deltoid and biceps weakness that partially recovered at 24 months [14]. These cases highlight the importance of a staged surgical strategy to maximise resection while minimising traction-related injury and postoperative neurological morbidity, particularly for tumours involving functional cervical roots (C5–T1) [20].

A six-month interval between procedures permitted complete wound healing, dural maturation, and confirmation of full neurological recovery to establish a clear baseline for the second operation, while enabling interval imaging to rule out delayed complications such as CSF leakage, haematoma, or cord oedema. This period also provided sufficient psychological and physical recovery before the second major procedure. Given the indolent growth of WHO grade I meningiomas, the risk of interval tumour progression was minimal. The present patient experienced no postoperative complications, including neurological deterioration or CSF leakage.

In conclusion, this case highlights the exceptional histopathological heterogeneity that can occur within dumbbell cervical meningiomas, with distinct transitional and fibrous subtypes identified in the intradural and extraforaminal components, respectively. Successful total resection was achieved using a tailored two-stage surgical strategy, which resulted in excellent long-term neurological and oncological outcomes. Our case underscores the importance of recognising possible histological diversity in dumbbell-shaped meningiomas and adopting individualised surgical strategies to optimise outcomes in these rare and challenging lesions.

## Supplementary Information

Supplementary Material 1.

## Data Availability

All data generated or analysed during this study are included in this published article.

## Abbreviations

CSF: Cerebrospinal fluid
CNS: Central nervous system
GTR: Gross-total resection
MRI: Magnetic resonance imaging
WHO: World Health Organization
